# Advances in mechanotransduction and sonobiology: effects of audible acoustic waves and low-vibration stimulations on mammalian cells

**DOI:** 10.1007/s12551-024-01242-1

**Published:** 2024-10-07

**Authors:** D. del Rosario-Gilabert, A. Valenzuela-Miralles, G. Esquiva

**Affiliations:** 1 Department of Optics, Pharmacology and Anatomy, University of Alicante, San Vicente del Raspeig, Spain; 2 Department of Physics, Systems Engineering and Signal Theory, University of Alicante, San Vicente del Raspeig, Spain; 3 Department of Computer Technology, University of Alicante, San Vicente del Raspeig, Spain; 4 Institute for Advanced Neuroscience of Barcelona (INAB), Barcelona, Spain; 5 Instituto de Investigación Sanitaria y Biomédica de Alicante (ISABIAL), Alicante, Spain

**Keywords:** Mechanotransduction, Sonobiology, AAW, LVS

## Abstract

In recent decades, research on mechanotransduction has advanced considerably, focusing on the effects of audible acoustic waves (AAWs) and low-vibration stimulation (LVS), which has propelled the field of sonobiology forward. Taken together, the current evidence demonstrates the influence of these biosignals on key cellular processes, such as growth, differentiation and migration in mammalian cells, emphasizing the determining role of specific physical parameters during stimulation, such as frequency, sound pressure level/amplitude and exposure time. These mechanical waves interact with various cellular elements, including ion channels, primary cilia, cell–cell adhesion receptors, cell–matrix and extracellular matrix proteins, and focal adhesion complexes. These components connect with the cytoskeletal fibre network, enabling the transmission of mechanical stimuli towards the nucleus. The nucleus, in turn, linked to the cytoskeleton via the linkers of the nucleoskeleton and cytoskeleton complex, acts as a mechanosensitive centre, not only responding to changes in cytoskeletal stiffness and nuclear tension but also regulating gene expression through the transcriptional co-activator YAP/TAZ and interactions between chromatin and the nuclear envelope. This intricate chain of mechanisms highlights the potential of sonobiology in various fields, including dentistry, regenerative medicine, tissue engineering and cancer research. However, progress in these fields requires the establishment of standardized measurement methodologies and biocompatible experimental setups to ensure the reproducibility of results.

## Introduction

Upon re-entry to Earth’s atmosphere following extended periods in outer space, astronauts exhibit symptoms of muscle atrophy, immune system dysfunction and bone demineralization (Hawkey 2003; Payne et al. 2007). As a result of exposure to microgravity, individuals experience gravitational forces which impact the function of their organs, cells and tissues. Living organisms are subject to a variety of physical factors including atmospheric pressure, hydrostatic pressure, gravity and mechanical or vibrational waves (Huang et al. 2013a; Ingber 2003). These forces, though appearing benign, are essential for the preservation of the organization and operation of cells, tissues and organs (Huang et al. 2013a).

Mechanotransduction is the study of how cells convert biophysical signals from the extracellular environment into intracellular biochemical signals and its biological implications for the human body (Dupont et al. 2011; Song et al. 2020). Mechanotransduction is a fundamental process observed in organisms starting from the initial phases of embryonic growth (Farge 2011). It plays a crucial role in maintaining homeostasis, promoting tissue regeneration (Tassinari et al. 2023) and contributing to the development of disease (Tortorella et al. 2022). Its applications in medicine demonstrate potential for the treatment of bone diseases (Jepsen et al. 2017; Li et al. 2021), tissue engineering (Tsata and Beis 2020), tumour development (Blanco et al. 2023; Vasilaki et al. 2021) and chronic pain (Ma and Quirion 2008; St-Jacques and Ma 2014).

For all these reasons, there has been a growing interest in the scientific community in recent years. In 2022, mechanobiology had accumulated over 64,226 publications (data calculated using keywords from Wall et al. (2018) and limiting the Web of Science search to the categories of Biochemistry Molecular Biology, Biology, Biophysics, Biotechnology Applied Microbiology, Cell Biology or Cell Tissue Engineering (Wall et al. 2018)). To date, significant advances have been reported in the prevention, diagnosis and treatment of pathologies related to ageing, cancer and other dysfunctions affecting various tissues and organs (Stylianopoulos et al. 2012; Wells 2013).

In this context, it is recognized that cells possess the ability to sense diverse mechanical cues originating from the extracellular matrix (ECM) and transduce them into intracellular biochemical signalling events that modulate cellular behaviour and functionality. The mechanical stimuli present in the ECM can manifest as either static or dynamic forces (Bukoreshtliev et al. 2013). Various types of mechanical stimulus, including compressive forces, tension and shear, have been extensively studied in scientific literature (Cai et al. 2021; Le et al. 2020). Despite evidence demonstrating the impact of audible acoustic waves (AAWs) within the 20 Hz–20 kHz range on cells, the ultrasonic range has received the most research attention (Havelka et al. 2011). Sonobiology is the scientific discipline focused on the study of mechanotransduction processes initiated by AAWs and their associated vibrations. This work elucidates the biophysical principles underlying sonobiology, its primary biological impacts on mammalian cells, the identified mechanotransduction events and the potential applications of this field in biomedicine.

## Biophysical foundations of sonotransduction

Physically, an AAW is a fluctuation in dynamic pressure that travels through a medium with specific properties including temperature, humidity, density and elasticity (Beranek and Mellow 2012). Sound waves can be classified as either simple or complex signals depending on their spectral characteristics. Simple signals, such as sine waves, display properties such as waveform, amplitude and frequency. In contrast, complex waveforms are characterized by their amplitude and spectral composition. Furthermore, when a rigid object is exposed to an acoustic wave, it responds to changing pressures through vibrations. These vibrations involve the propagation of wave energy within the object, leading to mechanical oscillations (Doyle 2021; Fahy and Gardonio 2007).

**Fig. 1:**
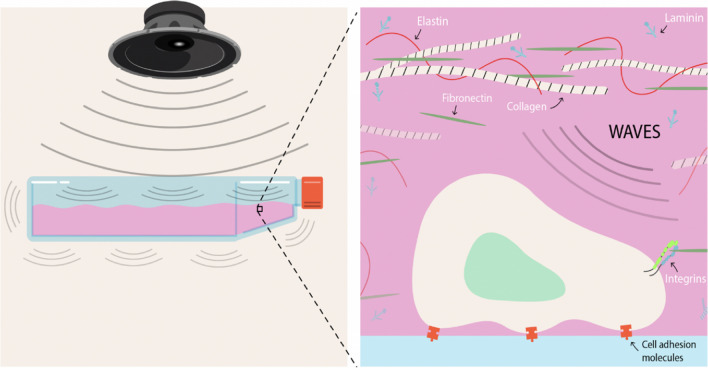
Cellular stimulation by acoustic waves. When an acoustic wave interacts with a biological culture plate, two main phenomena occur: (i) a considerable amount of energy is transmitted through the material, and (ii) the wave–culture plate interaction vibrates the structure. On the one hand, the energy transmitted through the culture plate reaches the extracellular matrix, a dynamic 3D network of proteins, glycoproteins, proteoglycans, glycosaminoglycans and hyaluronan (elastin, fibronectin, collagen and laminins among others). At the same time, the vibrational energy induced by the wave–culture plate interaction affects the cellular environment and the adhesion molecules that keep the cell attached to the substrate. These changes in the cellular environment could induce specific intracellular responses

In the field of sonobiology, multiple phenomena are observed when an AAW interacts with a biological culture plate. Analysis from a wave perspective revealed that a considerable amount of the wave’s energy is either reflected or transmitted through the material. The application of AAWs results in the initiation of vibrations within the culture plate, leading to displacement of its particles from their resting state. The specific modes of vibration produced are determined by the plate’s geometric shape and size and the constraints imposed by its boundaries (Doyle 2021). As a result, the stimulation within the ECM that initiates sonotransduction is a combination of the energy propagated through the culture vessel and its modes of vibration (Fig. 1).

### AAW propagation in liquid

Sound waves propagate at a greater velocity in a liquid medium, such as water, in comparison to a gaseous medium, such as air (Durá 2005). When studying the transmission of sound waves in the ECM, it can be compared to the analysis of a mechanical wave in a liquid medium under certain assumptions. A liquid medium in thermodynamic equilibrium can be characterized by its density (${\rho }_{\text{m}}$), static pressure ($P$) and temperature ($T$), which are constant. In cases where the liquid has high thermal conductivity, the temperature variations within the medium are negligible, leading to a simplification of the equations of state.

1 $${\beta }^{p}=\frac{1}{{\rho }_{\text{m}}}{\left(\frac{\delta {\rho }_{\text{m}}}{\delta P}\right)}_{T}$$

where ${\beta }^{p}$ is the isothermal compressibility coefficient, and $\delta {\rho }_{\text{m}}$, $\delta T$ and $\delta P$ represent the instantaneous incremental variations in density, temperature and pressure caused by the sound wave, respectively (Dukhin and Goetz 2002). In a broad context, the pressure fluctuation is described by the three-dimensional wave equation in a uniform and homogeneous liquid (Llinares Galiana et al. 1996):

2 $${\nabla }^{2}p=\frac{1}{{v}_{\text{m}}^{2}}\frac{{\delta }^{2}p}{{\delta t}^{2}}$$

where ${\nabla }^{2}=\frac{{\delta }^{2}}{{\delta x}^{2}}+\frac{{\delta }^{2}}{{\delta y}^{2}}+\frac{{\delta }^{2}}{{\delta z}^{2}}$ and the speed of sound in the medium (${v}_{\text{m}}$) is expressed as:

3 $${v}_{\text{m}}^{2}=\frac{1}{{\beta }^{p}{\rho }_{\text{m}}}$$

In this context, wave Eq. (2) has a general solution of the form:

4 $$p(\overrightarrow{r},t)=A{e}^{i\left(\overrightarrow{k}\overrightarrow{r}-\omega t\right)}$$

where $\overrightarrow{r}=\left(x,y,z\right)$, the pulsation $\omega =v\left|\overrightarrow{k}\right|$ and the wavenumber $\left|\overrightarrow{k}\right|=\sqrt{{k}_{x}^{2}+{k}_{y}^{2}+{k}_{z}^{2}}$.

Ultimately, the specific acoustic impedance is a critical parameter that characterizes the properties of a medium and governs the connection between acoustic pressure and particle velocity at a given spatial point:

5 $$Z=\frac{p}{{v}_{\text{m}}}$$

### Vibration of structures containing fluids

When an acoustic stimulus is reached to a culture plate, it not only travels through the liquid medium but also interacts with the culture vessel housing the cells and the cell medium. The presence of cells and medium acts as a ‘virtual mass’ within the culture vessel, impacting its natural frequencies and vibrational patterns (Kwak 1996). The subsequent discussion focuses on analysing the influence of liquid on the vibrational modes of a rectangular container.

A uniform, flat, horizontal plate is observed to exhibit buoyancy when placed on the surface of a homogeneous, incompressible liquid (Robinson and Palmer 1990). The plate’s surface in contact with the gaseous medium is subjected to a pressure, denoted as $p(x,t)$, while the surface in contact with the liquid experiences a pressure denoted as $p^{\prime} (x,t)$. Classical theory posits that, under small amplitude displacements, the stress distribution within a plate is negligible and the plate’s mechanical response is primarily influenced by:

6 $$m\frac{{\delta }^{2}y}{{\delta t}^{2}}+c\frac{\delta t}{\delta y}+\frac{E{h}^{4}}{12\left(1-{v}^{2}\right)}{\nabla }^{4}y={p}^{\prime}\left(x,t\right)-p\left(x,t\right)$$

where $x$ represents the horizontal coordinates, $y$ the vertical displacement, $t$ the time, $m$ the mass per unit area of the plate, $c$ the damping force per unit area per unit velocity, $E$ the Young’s modulus of the material, $v$ the Poisson’s ratio and $h$ the thickness of the plate.

In summary, the mathematical model assumes the following: (i) the fluid is homogeneous, incompressible, non-viscous and irrotational, with a density ($\rho$) and a constant depth ($d$); (ii) the weight of the fluid is dominant compared to the weight of the plate, leading to low amplitude oscillations and low particle velocities; and (iii) the displacement of the fluid surface is significantly less than the depth.

Applying Bernoulli’s transient equation, the pressure at the fluid surface can be calculated by the following mathematical expression:

7 $$-\rho {\left.\frac{\delta \phi }{\delta t}\right|}_{z\approx 0}+{p}^\prime+\rho gy=0$$

By integrating the equations governing the plate (6) with the equation describing the influence of a liquid (7) on one of its faces, a comprehensive equation for the studied system is derived.

8 $$-{\left.\frac{\delta \phi }{\delta z}\right|}_{z\approx 0}\approx\frac{\delta y}{\delta t}$$

The solution to Eq. (8) enables modal analysis of the system.

9 $$\left({\nabla }^{2}-\mu \right)\left({\nabla }^{2}+\mu \right)\psi \left(x\right)=0$$

where $\psi \left(x\right)$ describes the horizontal spatial variation of $y$ and $\mu$ is a constant.

By applying the boundary conditions to a rectangular plate with dimensions ${L}_{1}$ × ${L}_{2}$ and a liquid of depth ($d$), an expression for calculating the eigenmodes is derived:

10 $${\psi }_{jk}\left(x\right)={a}_{jk}\text{cos}\left(\frac{j\pi {x}_{1}}{{L}_{1}}\right)\text{cos}\left(\frac{j\pi {x}_{2}}{{L}_{2}}\right)$$

**Fig. 2:**
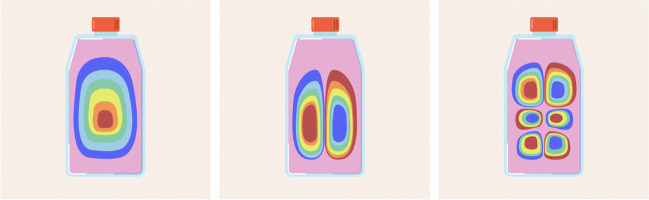
Simplified eigenmodes of vibration of a cell culture flask. The first vibration mode, the third vibration mode and the sixth vibration mode of a rectangular plate in contact with water on one side (50% depth) are shown. The red colour represents the maximum vibration energy, and blue represents the minimum vibration energy. The presence of a fluid acts as a virtual mass that influences its natural frequencies and vibrational patterns

Multiple theoretical frameworks have been suggested in scholarly works, including the Mindlin theory, the Rayleigh–Ritz theory and the Bernoulli–Euler model (Doyle 2021; Khorshidi et al. 2017). Hence, it is viable to exhibit the eigenmodes of vibration of an immobile rectangular plate in contact with a liquid on one side (Fig. 2).

In addition to the classical models, the study of eigenmodes of cell culture elements, such as a standard 96-well plate with complex stress distributions, is possible through the use of a computational model based on finite element methods (FEMs) (Matsui et al. 2018).

## Biological effects of sonotransduction

In the field of biomedical research, the integration of engineering principles, clinical interventions and mechanobiology is essential for understanding cell behaviour, differentiation and response to external stimuli. When an AAW reaches a cell culture support, a portion of the energy emitted is directly transmitted to the biological system, causing a wave–cell interaction. Another portion of the energy causes vibration in the structure, resulting in a structure–cell interaction and ultimately leading to low-vibration stimulation (LVS). Following a biophysical analysis (refer to subsections ‘AAW propagation in liquid’ and ‘Vibration of structures containing fluids’), we examine research on vibrational sonotransduction and experimentation with loudspeaker-based electroacoustic systems and investigate their physiological effects on animal cells.

### LVS on mammalian cells

The inside of the culture plate presents an air–liquid interface. Vibrations can be generated in an LVS in different ways. The most prevalent involves the utilization of a standard transducer affixed to the plate, bioreactors or piezoelectric actuators. The study by Mohammed et al. (2016) demonstrated that loudspeaker-based systems can achieve working frequencies ranging from 20 to 1600 Hz. Alternatively, bioreactors utilizing a piezoelectric vibrating plate have been employed. In this configuration, the culture material is affixed magnetically to the plate, which vibrates in response to an input voltage signal at the nanometre scale, operating within a frequency range of 1000–4000 Hz (Tsimbouri et al. 2017). Systems operating at frequencies in the order of kilohertz utilize piezoelectric actuators that are stimulated by an alternating current, resulting in periodic expansion and contraction of the actuator. This vibration is then transferred to the culture plate (Ambattu and Yeo 2023; Enomoto et al. 2020). Next, a summary table outlining the primary vibroacoustic stimuli in mammalian cells is provided (Table 1), followed by an analysis of key research findings from recent decades.

**Table 1:** Applied vibroacoustic research in mammalian cells

Vibroacoustic stimuli			Cell type	Biological technique	Summary
Frequency	Amplitude	Time			
5–2000 Hz	7.838* g* and 4.098* g*	2 min, 6 min and 30 min	Mouse osteoblast cells (MC3T3E1)	RT-PCR and immunoassay	The alterations in messenger ribonucleic acid (mRNA) expression are attributed to a direct mechanical influence of the vibrational force (Tjandrawinata et al. 1997)
20 kHz	1.5 W	15 s	Bovine aortic smooth muscle cell (SMC)	Tetrazolium (MTT) assay, trypan blue exclusion assay	The vibrational forces inhibit the adhesion and migration processes (Alter et al. 1998)
60 Hz	0.3* g*/50 µm	1 h/day for 4 non-consecutive days	Rat bone marrow–derived mesenchymal stem cells (rBM-MSCs)	Proliferation assay, quantitative PCR (mRNA), ALP assay and quantification of matrix mineralization	Cell proliferation was observed to increase, resulting in a decrease in osterix mRNA levels and inhibition of matrix mineralization (Lau et al. 2011)
90 Hz	0.7* g*	Between 20 min/day and 6 h/day for 7–8 days	Fibroblast of C3H mouse embryo cells (C3H10T1/2-MSCs)	Quantitative reverse transcription polymerase chain reaction (RT-qPCR), western blotting and histochemical staining	2 20-min periods of LVS inhibited adipogenesis (Sen et al. 2011)
25 Hz, 50 Hz, 100 Hz, 500 Hz and 1000 Hz	10–20 V	7 days	Human mesenchymal stem cells (hMSCs)	Immunofluorescence, PCR and microarray	Enhancement of *RUNX2* gene expression through 1 kHz LVS (Nikukar et al. 2013)
30 Hz, 400 Hz and 800 Hz	0.3* g*	30 min/day for 14 days	Human bone marrow–derived mesenchymal stem cells (hBM-MSCs)	Cell viability stain and cell proliferation assay, total RNA extraction and RT-qPCR	Improved osteogenesis with 800 Hz LVS and vibration at 30 Hz supported adipogenesis (Chen, X. et al. 2015)
1 kHz, 2 kHz and 3 kHz	20 V, 3.6* g* and 20* g*	21 days	hMSCs	Immunofluorescence, western blot, RT-qPCR, Raman spectroscopy, Alizarin Red S staining and Von Kossa staining	Optimal osteogenesis at 1000 Hz using LVS (Pemberton et al. 2015)
100 Hz, 200 Hz, 400 Hz, 800 Hz and 1600 Hz	0.2 W	5 min	Human lung fibroblasts (LL24), mouse fibroblasts (L929)	MTT	Migration distance was found to be dependent on the frequency/amplitude of LVS. Specifically, a frequency of 100 Hz was found to result in an increase in migration distance (Mohammed et al. 2016)
40 Hz	0.3* g*	15 min/day for 14 days	rBM-MSCs	Oil Red O staining, RT-qPCR, western blot and immunofluorescence staining	LVS promotes adipogenesis and enhances osteogenesis (Zhao et al. 2017)
1 kHz	30 nm and 80 V	21 days	hBM-MSCs	Interferometric measurement, characterization of collagen matrices by AFM, alamarBlue assay, live–dead staining, inhibition assays, western blot, RT-qPCR, Raman spectroscopy, Von Kossa staining, micro-CT (µCT) analysis and metabolomics	LVS stimulates osteogenesis (Tsimbouri et al. 2017)
1 kHz	22 nm	7 days	Human mesenchymal stem cell	Osteogenesis measurement by Von Kossa staining, RT-qPCR	LVS stimulates osteogenesis (Robertson et al. 2018)
20 Hz, 60 Hz and 80 Hz	2.173* g*	Unspecified	Immortalized mouse olfactory ensheathing cells (OECs)	Spheroid-producing naked liquid marble (NLM) technique	Better migration response at 60 Hz VLS (Beckingham et al. 2019)
1 kHz	30 nm and 90 nm	9 days	Stro-1-selected MSCs from adult human bone marrow	Scanning electron microscopy, composite contraction measurement and time lapse microscopy, cryosection and immunostaining, interferometric measurement, alamarBlue assay, RT-qPCR, inhibitor studies, protein antibody microarrays, metabolomics, reactive oxygen species measurement, and ELISA	Higher amplitude of VLS increases osteogenesis and ion channel expression (Orapiriyakul et al. 2020)
11.2 kHz	2 V and 4 V	24 h	Mouse fibroblasts (L929)	Collective cell migration experiment, glucose consumption assay, cell staining and image‐based quantification	Direction of vibration affects cell migration distance (Enomoto et al. 2020)
1 kHz	40.6 nm and 44.4 nm	7 days	Primary human osteoprogenitor and osteoclast progenitor cell co-culture (human bone marrow haematopoietic cells, osteoclast precursors, mesenchymal stromal cells, osteoprogenitors, osteoblasts and osteocytes)	3D cell culture technique, TRAP staining, immunostaining, Von Kossa staining, scanning electron microscopy, alamarBlue assay, RT-qPCR, ELISA, osteoclast functional assessment and metabolomics	LVS inhibits osteoclast differentiation in CD14^+^ monoculture, upregulation of osteogenesis and inhibition of osteoclastogenesis in BM-MSC/BMHC co-culture (Kennedy et al. 2021)
100 Hz, 500 Hz and 1000 Hz	0 to 24 V	5 min	Murine adherent cell lines (L929, HeLa and MG-63)	Cell viability, cell proliferation rate and cytotoxicity (alamarBlue^©^ assay), cell morphology, cell membrane permeability, physicochemical characterization of polyplexes, luciferase assay substrate, RT-qPCR and protein expression	Proposes the use of LVS to enhance the efficiency of gene delivery vectors in vitro and ex vivo environments (Ponti et al. 2022)

From left to right, we find the first section, ‘vibroacoustic stimuli’, where the physical characteristics of the acoustic waves used in the studies (frequency and amplitude), as well as the exposure time of the stimulus to the cells, are presented. Next, we find the cell lines used, the biological techniques employed and the main findings reported in the studies.

The study of Sen et al. (2011) delves into LVS on mesenchymal stem cell (MSC) fate, focusing on their impact on adipogenesis. Their work emphasizes that the effect of LVS on mesenchymal stem cell lineage selection is primarily influenced by the timing of the events rather than the intensity or duration of the load. Introducing multiple short mechanical challenges over a 24-h period may enhance beneficial outcomes in living organisms. Ultimately, the research highlights that incorporating refractory periods between mechanical loading bouts enhances the LVS ability to inhibit adipogenesis and suggests that event scheduling may be more critical than load magnitude or duration in influencing MSC lineage selection, offering insights for potential future therapeutic strategies (Sen et al. 2011).

The study conducted by Chen et al. (2015) examines the effects of LVS on the differentiation of human bone marrow–derived mesenchymal stem cells (BM-MSCs) towards osteogenesis and adipogenesis. It found that different frequencies of LVS induced frequency-dependent responses in cell behaviour, with 800 Hz LVS being the most favourable for osteogenic differentiation while simultaneously suppressing adipogenesis. The authors highlight the significance of maintaining the balance between osteogenesis and adipogenesis in order to maintain bone homeostasis and propose that LVS may modulate lineage-specific differentiation in BM-MSCs. The findings suggest that LVS could potentially be a novel approach for preventing and treating osteoporosis (Chen et al. 2015).

Mohammed et al. (2016) investigated the effects of LVS on the migratory and morphological properties of two different fibroblast cells: human lung fibroblast cells (LL24) and subcutaneous areolar/adipose mouse fibroblast cells (L929). The study used a speaker-based system to apply LVS at frequencies from 0 to 1600 Hz for 5 min and examined the impact on cell migration distance and actin organization. The results showed that 100 Hz LVS enhanced cell migration for both cell lines, while frequencies above 100 Hz decreased cell migration in a frequency-dependent manner. Additionally, mechanical stimulation promoted changes in cell morphology, particularly the formation of lamellipodia and filopodia, with different prominence in the two cell lines. The study suggests that LVS may be used to manipulate the mechanosensitivity of cells to promote cell migration. This preliminary work highlights the potential of using mechanical stimulation to enhance the wound healing (Mohammed et al. 2016).

In the study conducted by Zhao et al. (2017), the effects of LVS on adipogenesis in BM-MSCs were investigated. The study utilized LVS at 0.3* g*, 40 Hz and 50 µm amplitude for 15 min daily. The research aimed to understand the molecular mechanisms underlying LVS influence on adipo-differentiation of BM-MSCs. It was found that LVS increased the expression of adipogenic markers such as PPARγ, C/EBPα and adiponectin, as well as the formation of oil droplets in BM-MSCs. The study also revealed the involvement of the p38 mitogen-activated protein kinase (p38 MAPK) signalling pathway in LVS-induced adipogenesis. Activation of p38 MAPK was observed after treatment, and inhibition of p38 MAPK resulted in decreased expression of adipogenic markers and reduced oil droplet formation. The study provides valuable insights into the impact of LVS on adipogenic differentiation of BM-MSCs and the associated molecular mechanisms (Zhao et al. 2017).

Enomoto et al. (2020) explored the effect of vibration direction on the collective migration of fibroblasts in a wound model. A vibrating system was developed to apply horizontal LVS to a cell culture dish, and its effects on cell migration distance, glucose consumption and cell nuclei orientation were evaluated. It was observed that the vibration direction significantly affected cell migration distance, with orthogonal vibration enhancing migration and parallel vibration suppressing it. Glucose consumption was elevated under conditions that promoted migration distance, indicating increased energy consumption for cell migration. Cell nuclei became elongated and oriented orthogonal to the gap under conditions promoting migration, while parallel vibration resulted in cell nucleus orientation in the direction parallel to the gap. The study suggests that horizontal vibration can regulate collective cell migration and provides new insights into mechanotransduction in cell migration (Enomoto et al. 2020).

The study of Kennedy et al. (2021) explores the use of a bioreactor to inhibit osteoclast formation and activity while enhancing osteogenesis in a co-culture of primary human osteoprogenitor and osteoclast progenitor cells. Specifically, this study focuses on the effects of 1000 Hz frequency and 40 nm amplitude vibration on osteoclast formation and activity in CD14^+^ human mononuclear blood cells. The research introduces the potential of LVS as a method for promoting bone regeneration and reducing osteoclast activity. This research suggests that LVS could be utilized to create humanized 3D models for drug screening in the field of osteoporosis and bone homeostasis research. These findings have potential implications for clinical applications and drug discovery in the future (Kennedy et al. 2021).

The work of Ponti et al. (2022) introduces a gene delivery strategy termed ‘Vibropolyfection’ that greatly enhances the efficiency of non-viral vector transfection. The study describes the application of an LVS to adherent cells, leading to increased transfection efficiencies for nucleic acid/polymer nanoparticles, without compromising cell viability or proliferation. This novel approach has the potential to significantly enhance the efficacy of gene delivery vectors in vitro and ex vivo, representing a potential breakthrough in molecular medicine and advanced therapeutics (Ponti et al. 2022).

In conclusion, the findings presented emphasize the significant impact of LVS parameters such as amplitude, frequency and timing on diverse mammalian cellular processes. The frequency-dependent responses, amplitude-mediated cellular differentiation and the potential significance of event scheduling in influencing cell fate allocation highlight the intricate interplay between LVS and mammalian cellular behaviours. These insights offer valuable pathways for understanding and manipulating mammalian cellular responses, holding great promise for future developments in clinical interventions, regenerative medicine and tissue engineering.

#### Aaws on mammalian cells

The methodologies utilized in the recent research in the field of sonotransduction involve radiating AAW stimuli and studying their impact on cell behaviour. These methods can vary significantly in terms of experimental set-ups, including a wide range of frequencies and waveforms (ranging from pure tones to broadband signals), different pressure levels (ranging from 20 to 100 dB) and exposure times (ranging from seconds to 3 days). Additionally, various cell lines are employed to investigate the effects of AAWs on cell behaviour. A tabular presentation detailing the main stimuli employed, cell lines utilized, biological methodologies employed and resultant conclusions is presented in Table 2.

**Table 2:** Sonotransduction research applying AAWs in mammalian cells

Acoustic stimuli			Cell type	Biological technique	Summary
Frequency	Sound pressure level/amplitude	Time			
261 Hz (sine tone)	87 dB	0 s, 15 s, 30 s, 60 s or 120 s (non-consecutive days)	Human gingival fibroblast cell	Coulter Cell Counter	Significant effects on cell proliferation were observed for AAWs lasting between 30 and 120 s in duration (Jones et al. 2000)
20–20,000 Hz (broadband music)	70–100 dB	30 min	Human cell (MCF-7 and MDA-MB-231)	Flow cytometry	AAWs can alter cellular morpho‑functional parameters (Lestard and Capella 2016)
1000 Hz (sine tones)	81 dB	7 days	Human mesenchymal stem cells	Neural differentiation (hBM-MSCs), colorimetric assay, western blot assay and immunocytochemistry	AAWs induce neuronal differentiation (Choi et al. 2016)
528 Hz (sine tones)	80 dB, 100 dB and 120 dB	1 h, 2 h, 4 h and 12 h	Human astrocyte cells	MTT, LDH and ROS assays	AAWs increased cells viability about 20% in ethanol-treated cultures (Babayi and Riazi 2017)
55 Hz, 110 Hz and 4000 Hz (sine tones), 400 Hz (triangle and square tones), and white noise	76 dB, 82 dB, 88 dB and 94 dB	120 min	Murine ST2 and bone marrow cells	RT-qPCR	The waveform of AAWs is a determinant in gene regulation. Significant suppression of target genes for white noise (Kumeta et al. 2018)
20–2000 Hz (broadband music, traffic noise and human voice)	60 dB	20 min	Murine atrial cardiomyocyte cells	Immunofluorescence, contractility and multifractal analysis	Different AAWs seem to influence the growth or death of cells (Lin et al. 2021)
150 Hz	20 µm, 40 µm, 60 µm, 80 µm and 100 µm	24 h, 48 h and 72 h	Human adenocarcinoma alveolar basal epithelial cells (A549)	Western blot, wound healing assay, Transwell migration and invasion assays, RT-qPCR, and protein analysis by LC–MS/MS	AAWs improve extracellular vesicle production (Lei et al. 2023)

From left to right, we have in the first section, ‘vibroacoustic stimuli’, the spectral characteristics of the audible acoustic waves used, as well as the exposure time of the stimulus to the cells. Next, we find the cell lines used in each study, as well as the biological techniques and the main findings reported.

Based on the collective findings, it is evident that an AAW has a significant impact on various types of cell in culture. The studies reviewed examined the effects of simple and complex AAWs on a range of cell types, including cancer cells, stem cells, cardiac muscle cells and gingival fibroblasts. The research highlights the influence of AAWs on cellular morpho-functional parameters, hormone binding, extracellular vesicle biofabrication, neural differentiation, contractility, spatial organization, gene suppression, proliferation and protein expression. Furthermore, the articles of greatest significance are thoroughly examined.

Lestard et al. (2013) investigated the impact of music on cellular morpho-functional parameters and hormone binding in a human breast cancer cell line (MCF-7). The study aimed to explore whether an AAW, specifically music, influences cellular behaviour in non-auditory cells. They exposed the cultured cells to different musical styles and frequencies, measuring parameters such as cell morphology, growth and hormone receptor expression. The research revealed notable variations in cellular responses to distinct musical genres, demonstrating that music exposure affected cellular functionality and hormone binding. The findings suggested a potential direct effect of music on non-auditory cells in culture, providing an insight into the interplay between AAWs and cellular behaviour (Lestard et al. 2013).

Choi et al. (2016) investigated the potential of AAW stimulation to induce neural differentiation in human BM-MSCs. The study aimed to understand the underlying mechanisms by which AAWs influence cellular behaviour and differentiation. The researchers exposed BM-MSCs to sound waves at 1000 Hz and 81 dB for 7 days, analysing the expression of neural markers and examining signalling pathways involved in the neural differentiation process. The findings revealed that AAWs induced neural differentiation of BM-MSCs and led to an increase in the phosphorylation of Pyk2 and CREB, key signalling molecules. Moreover, the study identified the involvement of ryanodine receptors and calcium release in sound wave–induced neural differentiation. These results suggest that specific AAW stimulation could serve as a potential inducer of neural differentiation in BM-MSCs (Choi et al. 2016).

Kumeta et al. (2018) investigated the impact of AAW as a mechanical stimulus for cells. The research focused on analysing gene responses to audible sound stimulation, examining various sound parameters such as frequency, waveform, composition and exposure time. The findings revealed a distinct suppressive effect on several mechanosensitive and ultrasound-sensitive genes triggered by AAWs, which varied based on waveform and pressure level, rather than frequency. The study identified two likely mechanisms involved in the cellular response to mechanical waves: transcriptional control and RNA degradation. Additionally, the research highlighted cell type–specific responses to AAWs, with different cell lines exhibiting varying degrees of sensitivity to audible sound stimulation (Kumeta et al. 2018).

Lin et al. (2021) explored the effects of AAWs on cardiac muscle HL1 cells. The study aimed to investigate how different stimuli influenced the contractility and spatial organization of HL1 cells. The researchers exposed the cells to different AAWs and analysed their contractility changes and spatial organization using advanced imaging techniques and multifractal analysis. The findings suggested that different AAW stimuli influenced the behaviour of HL1 cells, resulting in alterations in the spatial organization and contractility. The study also proposed a theoretical physical model to explain the observed cellular responses to AAWs, emphasizing the role of coherent molecular dynamics and the systemic view in understanding the biological activity of the mammalian cells (Lin et al. 2021).

Lei et al. (2023) explore a novel method to enhance the biofabrication of cancer-derived extracellular vesicles (EVs). The study aimed to investigate the impact of AAWs on the production and properties of cancer-derived EVs. The researchers exposed adherent cancer cells to AAWs and observed a significant increase in EV yield compared to conventional static culture, along with changes in EV morphology, size and zeta potential. Importantly, the EVs generated under AAW stimulation demonstrated the capability to promote cancer cell migration and invasion. Additionally, the study identified the activation of the ESCRT pathway and upregulation of membrane fusion–associated proteins in response to AAW stimulation. The findings suggest that an AAW represents a promising approach for enhancing the quantity and quality of EV production, with potential implications for advancing scientific and translational research involving EVs (Lei et al. 2023).

The synthesis of data underscores the pivotal role of amplitude, frequency and timing of AAWs on mammalian cells. The intricate interplay of these factors collectively governs the cellular responses to AAWs, elucidating the multifaceted effects on cellular behaviour and function. Moreover, the research underscores the significance of the timing and duration of sound exposure in producing consistent signals from various cytoskeletal components and modulating protein expression. This highlights the importance of the temporal dimension in the cellular response to AAWs.

The significance of these findings extends beyond the realm of basic science, emphasizing the need for meticulous optimization of AAW stimuli to harness their full potential for therapeutic and clinical applications. As previously observed, the AAW stimulation offers a promising avenue for the development of precise and targeted interventions, ultimately holding transformative potential for advancing biomedical research and improving patient care and outcomes.

### Need for experimental standardization and biocompatibility

While the research presented elucidates the significant impact of AAWs and LVS on cellular behaviour and highlights its potential for groundbreaking applications, it is evident that standardized experimental protocols and the use of biocompatible materials are crucial for advancing this field. As we consider the future of AAW stimulation in cell culture, the establishment of standardized measurement methods, controlled stimulation environments and reproducible experimental set-ups is imperative (Kwak et al. 2022). This will facilitate the comparability of results across studies, ensuring the reliability and validity of findings. Additionally, the utilization of biocompatible materials and platforms for delivering sound stimuli is paramount to minimize any potential adverse effects on cell viability and functionality. Addressing the issue of biocompatibility will facilitate the utilization of audible sound–based methodologies in a range of biomedical contexts.

## Mechanotransduction: from the ECM to the cell nucleus

The ECM is a dynamic three-dimensional network of macromolecules that, in addition to providing structural support, plays important roles in health and disease (Carmignac and Durbeej 2012; Iozzo and& Gubbiotti 2018). In essence, the ECM constitutes an organized network of proteins and glycoproteins (collagens, elastin, laminins and tenascins), proteoglycans, glycosaminoglycans and hyaluronan, which communicate with surrounding cells through cell surface receptors (Karamanos et al. 2021). Beyond the biochemical world, the cellular environment is a place replete with biophysical signals capable of inducing intracellular responses (Song et al. 2020). The molecules within the ECM network are physically connected from the ECM to the cytoskeleton and nucleoskeleton. Starting from the exterior of the cell, various ECM molecules such as fibrinogen and fibronectin are recognized by receptors on the cell surface. These molecules then bind to integrins, which serve as the primary connectors between the ECM and the cell in ECM adhesions. Integrins, in turn, are connected to the actin cytoskeleton through proteins that act as cross-linkers, playing a crucial role in mechanotransduction within ECM adhesions. Some of these proteins possess binding sites for both integrins and actin at different locations, facilitating the individual cross-linking of integrins and actin. Others lacking specific binding sites for integrins, or actin cross-link these components in a cooperative manner. The cytoskeletal filaments, including actin, intermediate filaments and microtubules, along with their associated cross-linkers, are all mechanically linked and thus participate in cell mechanotransduction. Finally, we find structures such as the linkers of the nucleoskeleton and cytoskeleton (LINC) complex, which play a crucial role in this process, as they allow the integration and transmission of signals to and from the cell nucleus, where a final response is formulated and executed.

A growing number of studies highlight how biophysical signals play a remarkable role in both general aspects of cell behaviour, such as cell adhesion, spreading, migration, growth and differentiation (Xie et al. 2023), and specific aspects, such as leukocyte extravasation, macrophage polarization, T cell selection and activation (Zhang et al. 2020). Broadly speaking, mechanotransduction, which is to translate the mechanical signal into biochemical pathways, can be divided into two groups: (i) proximal mechanotransduction and (ii) nuclear envelope receptors. While different cell typologies exhibit common mechanotransduction strategies, others seem to develop specialized adaptive strategies to specific mechanical forces (Kamkin and Kiseleva 2005; Ogawa 2016). Fibroblasts and cardiomyocytes are highly sensitive to stretch forces (Huang et al. 2013b; Murata et al. 2014), osteocytes to compressive forces or hydrostatic pressure (Huang and Ogawa 2012; Klein-Nulend et al. 1995) and chondrocytes to shear forces (Lee et al. 2002), and gastrointestinal smooth muscle cells are sensitive to myogenic reflex contractions (Joshi et al. 2021), inner ear hair cells to mechanical waves in the audible range (Vollrath et al. 2007) and sensory neurons to vibration or pressure (Tsunozaki and Bautista 2009). However, the scope of this article is limited to the most common mechanotransduction events in non-specialized cells, starting from proximal mechanotransduction (i) and then addressing nuclear envelope receptor types (ii).

### Proximal mechanotransduction: mechanical links between the extracellular matrix and the actin cytoskeleton

**Fig. 3:**
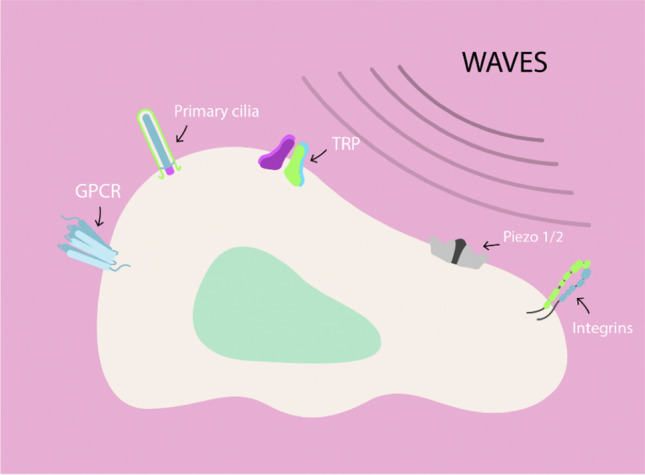
Key cellular components in proximal mechanotransduction. Cells have various mechanosensitive structures capable of integrating mechanical waves from the extracellular medium to their interior. Integrins stand out as transmitters of signals between the extracellular environment and the cell interior, bidirectionally. Furthermore, cells have various mechanosensitive channels such as members of the transient receptor potential vanilloid 4 (TRPV) and Piezos1/2 families that act by generating electrochemical gradients and triggering multiple biochemical signalling pathways. We also find primary cilia, sensory structures that protrude from the cell surface, capable of detecting mechanical forces and inducing intracellular signals that regulate gene expression

Proximal mechanotransduction encompasses those mechanotransduction events employed by the cell to convert available mechanical energy from the ECM into biochemical energy within the cell. Among the most studied mechanisms are, but are not limited to, ion channel proteins, cell–cell adhesion receptors (cadherins, intercellular adhesion molecule (ICAM)), cell–matrix adhesion proteins (integrins), extracellular matrix proteins (fibronectin), as well as the actomyosin cytoskeleton and focal adhesion complexes, notable for carrying out the transduction of biological signals from the ECM to the cell interior (Alenghat and Ingber 2002; Li et al. 2021; Wolfenson et al. 2019) (Fig. 3).

#### Mechanosensitive ion channels

Stress-activated (SA) membrane ion channels are ubiquitous mechanotransducers that support the conversion of mechanical force signals applied to the cell surface into transmembrane ion gradients (Matthews et al. 2006). Mechanosensitive ion channels are critical for cell survival and development in both eukaryotic and prokaryotic cells (Mount et al. 2022; Murthy et al. 2017). Ion current through mechanically activated SA channels is perhaps the fastest form of mechanotransduction, and the recent work suggests that integrins might leverage this mechanism for various types of physiologically relevant control mechanism (McMahon et al. 2008; Thodeti et al. 2009).

Mechanosensitive ion channels activated by the application of force to integrins (Matthews et al. 2007) induce a calcium influx into the cytoplasm, which can modulate contractility through calmodulin–caldesmon interactions and thereby actively influence both cytoskeletal organization (Sokabe et al. 1997) and cell stiffening behaviour (Helfman et al. 1999). Other studies suggest that the cell stiffening response can be influenced by the level of resting tension (pre-tension) in the cytoskeleton before the application of force (Stamenović and Wang 2011; Wang and Ingber 1994).

The recent research indicates that calcium entry through TRPV4 channels, found in the plasma membrane of bovine capillary endothelial cells, is essential for alterations in focal adhesion assembly, cell orientation and directional migration induced by mechanical tension. This localization is crucial for the mechanical regulation of several cell behaviours important for cellular and tissue development (Matthews et al. 2010).

Another important mechanosensitive ion channel is Piezo1. Due to its structural characteristics and the presence of blades embedded within the membrane, this channel exhibits a pronounced outward curvature in its closed state. This favours the transition from a bent to an extended state upon applying a maximum activation tension of 1.9 pN/nm to the membrane, opening the central pore and causing a non-selective cation flow (Yang et al. 2022). Additionally, the influx of ions such as Ca^2+^ following Piezo1 activation triggers multiple signalling pathways such as PI3K/Akt and membrane type 1-matrix metalloproteinase (MT1-MMP), implicated in processes like vascular remodelling and angiogenesis (Kang et al. 2019; Lai et al. 2021). Due to the importance of these pathways, various levels of regulation exist to modulate Piezo1 behaviour. For example, the loss of the actin cytoskeleton makes the channel more sensitive and easier to activate. In this case, the cytoskeleton acts as a membrane tension regulator, playing a mechanoprotective role and affecting Piezo1 activity (Cox et al. 2016). On the other hand, mechanosensitivity can be adjusted through counterinteraction between two and more channels (Brohawn et al. 2014). The TRAAK channel is part of a family of mechanosensitive potassium-selective channels (Enyedi and Czirják 2010). When activated through a membrane tension–dependent mechanism, it generates hyperpolarization in cultured N2A cells. This suppresses action potential activation in these cells by counteracting the depolarization caused by Piezo1 activation (Brohawn et al. 2014).

Overall, evidence demonstrates that mechanosensitive ion channels play a crucial role for cells to detect and respond to mechanical forces. Moreover, specific channels such as TRPV4 and Piezo1 regulate cell orientation, migration and vascular remodelling, exerting a significant influence on cellular and tissue physiology. Additionally, there is a sophisticated and multilevel regulatory system, such as counterinteraction between different channels or their relationship with the cytoskeleton, which helps to adapt the cellular response to various mechanical forces.

#### Primary cilia

Primary cilia act as mechanical sensors that detect physical forces, such as fluid flow or movement. Primary cilia are immobile, antenna-like organelles that protrude from the surface of most mammalian cells, capable of detecting mechanical forces and inducing intracellular signals that regulate gene expression (Delling et al. 2016; Eguether and Hahne 2018). Their basic architecture is composed of nine circular microtubule doublets or ‘axoneme’, formed by polymerized alpha and beta-tubulin dimers, and they extend as an independent unit from the basal body (Janke and Bulinski 2011). Additionally, they have specific ion channels located in the ciliary membrane that are activated when these cilia bend or move due to mechanical forces. Moreover, between the basal body and the cilium lies the ciliary transition zone, which contains specialized activation structures such as Y-links and transition fibres (Anvarian et al. 2019). This transition zone acts as a barrier that controls the lateral diffusion of membrane proteins between the cell body and the cilium (Park and Leroux 2022).

Functionally, primary cilia are involved in the development, organization, remodelling and function of various tissues. A classic example is the specification of the left–right body axis, allowing the development of asymmetrical organs such as the heart and lungs (Shinohara and Hamada 2017). Another example, at the cellular level, is their role in the process of osteogenesis. It has been shown that, after applying low-magnitude, high-frequency vibratory energy to osteoblasts, their differentiation is induced. The hypothesis is that these vibrations activate the COX-2/PGE/EP4 pathway in a primary cilia–dependent manner, and that these cilia are affected by this pathway (Haffner-Luntzer et al. 2018; Li et al. 2020).

Now, to analyse the functionality of primary cilia in terms of mechanosensation, the mechanical characteristics of these organelles must be investigated. The first representative model of the mechanical behaviour of the organelle, presented by Schwartz et al. (1997) under the framework of Euler–Bernoulli theory, assumes that the cilium behaves like a cantilever beam, protruding from the cell surface. Using this principle and employing various flow regimes, it was determined that the bending stiffness of the organelle was approximately 3.1 × 10^−23^ N/m^2^ (Schwartz et al. 1997). However, the simplicity of the model does not consider important factors such as basal rotational movements or hydrodynamic interactions. Therefore, improved models have emerged, such as the one proposed by Resnick (2015). Using a three-dimensional single-beam optical trap, they showed that, for cilia longer than 7 µm, the non-linear rotational spring behaviour of the base caused a change in the sign of the equilibrium deformation function slope (Resnick 2015). This supports the idea that certain structures located at the base of the cilium, such as the basal body and transition fibres, may have significant implications in the mechanosensation process. In line with this idea, there is research that considered the mechanical responses of the subaxonemal compartment. Using the Stokes flow regime to include the hydrodynamic interaction between the primary cilium and the flow, they calculated that the tensile force along the filament was not homogeneous, finding a maximum at the junction with the plasma membrane (Young et al. 2012). This has physiological implications, as it is more likely that simple mechanosensitive channels near the ciliary base are opened due to bending under flow.

Furthermore, the development of computational tools has enabled the generation of computational models to investigate the biomechanics of primary cilia. For example, using finite element analysis, the effects of 10% tensile stress applied to a primary cilium embedded in a collagen matrix were modelled (Mathieu et al. 2014). This study proposed a new view, where the orientation of the primary cilium may be more important than its length in the mechanosensation process. Additionally, they indicated that the amplification of tensile stress was concentrated around the ciliary base (Mathieu et al. 2014). However, another subsequent investigation, using an immersed boundary lattice Boltzmann method and considering the bidirectional fluid–structure interaction, found that, under pulsatile flow conditions, the maximum tensile stress of the cilium was not always in the basal region, but could propagate periodically at a certain distance (Cui et al. 2020). On the other hand, a multistructural cell model has also been developed to investigate the effect of ciliary biomechanics on various cellular components. The results of this study revealed that the deflection of the primary cilium under fluid flow surprisingly did not generate any significant effect on actin bundles and microtubules, although it did create greater stress transmission to the cell nucleus (Khayyeri et al. 2015). Additionally, they found that mechanical stress was transmitted to other organelles such as the Golgi complex (Khayyeri et al. 2015). This result is consistent with the hypothesis of the functional relationship between the primary cilium and the Golgi complex, where the cilium is involved in a functional feedback loop that collects extracellular signals, which are translated to the Golgi complex to facilitate targeted secretion of macromolecules (Poole et al. 1997).

Overall, the creation of representative models of primary cilia, whether based on in vitro experiments or through computational simulations, aims to characterize the biomechanical properties of this organelle. The goal of these models is to understand the functional role of primary cilia as mechanosensory organelles in the complex biological context.

#### Cell–ECM mechanosensitive molecules: fibronectin and integrins

Mechanotransduction events that occur within the cell begin at the membrane–ECM interface. Several ECM molecules are involved in cell–ECM adhesion. Here, the main ECM molecules that play a significant role in mechanotransduction are explained (Jahed et al. 2014).

##### Fibronectin

Fibronectin (FN), a glycoprotein found in connective tissues, is frequently associated with mechanotransduction processes. Structurally, each fibronectin typically presents in a dimeric form, with each monomer containing 3 repeating modules: 12 type I modules, 2 type II modules and 15 type III modules (Dalton and Lemmon 2021). Additionally, within the FN-III10 motif, it contains two arginine–glycine–aspartic acid (RGD) sequences that bind to various integrins, including those containing β1 and β3 (Humphries et al. 2006). Moreover, the RGD loop acts as a molecular sensor for traction force recognition, strategically located to undergo conformational changes from a β-turn to a linear conformation when tension increases above a threshold (Krammer et al. 1999). This results in a decrease in affinity and selectivity for integrins, directly impacting the cellular response to various stimuli such as mechanical waves.

Furthermore, significant force must be maintained between integrin and fibronectin to transmit a signal into the cell. From a biophysical perspective, the RGD–integrin interprotein bond exhibits catch-slip behaviour (Kong et al. 2009). In this type of interaction, the bond lifetime increases as the force applied to the integrin bound to the ligand increases, until reaching a maximum known as the optimal bond force. Conversely, when the applied force exceeds the optimal bond force, the bond lifetime rapidly decreases (Kong et al. 2009). Additionally, the RGD sequence is not the only component of fibronectin involved in integrin binding. In the FN-III9 module, fibronectin possesses the so-called ‘synergy site’, which is functionally decoupled from the RGD loop. Nonetheless, the relative distance between the two, about 32 Å under basal conditions, can affect the effective interactions between fibronectin and integrins, thereby impacting cell adhesion (Krammer et al. 2002).

In addition to its RGD domain that binds to cells, fibronectin also has domains that bind to type I collagen (col-1), facilitating the formation of networks composed of fibronectin and col-1 (Kubow et al. 2015). Unlike collagen and fibrin, fibronectin does not independently form fibrous networks nor respond to external chemical stimuli. Instead, cellular forces are required to assemble individual fibronectin molecules into insoluble elastic fibres, where cellular traction opens cryptic domains within fibronectin, mediating the formation of cross-links and networks (Baneyx et al. 2002).

Generally, due to the elastic and cohesive properties of fibronectin, it serves as a bridging link between the extracellular matrix and the cell through its interaction with integrins. This is why fibronectin coatings are commonly employed in 2D cell culture studies in the field of mechanotransduction, with the RGD motif being fundamental in synthetic cell culture methodologies based on ECM (Saraswathibhatla et al. 2023).

##### Integrins

Integrins are signalling proteins that facilitate bidirectional communication between the extracellular environment and intracellular pathways. Structurally, they are heterodimeric molecules that exist in at least 24 unique combinations of α subunits (18 types) and β subunits (8 types), interacting non-covalently. This configuration enables binding with a wide variety of ECM components and counter-receptors on other cell types (Bennett et al. 2009; Luo et al. 2007).

A crucial factor in evaluating the functionality of integrins is that they are not constitutively active. The activation of an integrin, transitioning from a low-affinity binding state to a high-affinity state, requires dynamic transitions from inactive bent conformations to active extended conformations (Kolasangiani et al. 2022). These long-range conformational changes can be triggered by cytoplasmic interactions (inside-out activation) (Tadokoro et al. 2003) or extracellular interactions (outside-in activation) (Puklin-Faucher and Vogel 2009).

##### Outside-in integrin activation

In outside-in integrin activation, external force is fundamental in regulating integrin functionality (Chen et al. 2017a). One variable with significant implications in the activation mechanism of integrins is ligand binding kinetics. It has been shown that for integrins α4β1 and α5β1 in a closed, low-affinity state, ligand binding activation rates are 40 times higher for α4β1 and 5 times higher for α5β1 compared to the extended, open conformation (Li et al. 2021). This finding has two important implications. First, most ligand binding in these representative β1 integrins occurs in a closed-bent state. Second, integrin–ligand binding can precede integrin activation, which is counterintuitive to the classical conceptual framework (Li et al. 2021).

Another aspect to consider in this outside-in mechanism is whether, once the ligand is bound to the integrin, external force could be sufficient to induce the necessary conformational changes leading to integrin activation. Various experiments with different approaches support this idea (Chen et al. 2017b, 2024). For example, molecular dynamics simulations demonstrated that the binding of the RGD motif of fibronectin to an αVβ3 integrin head induces the hinge angle opening between the integrin’s βA and hybrid domains. This hybrid domain swing is directly related to the transition of integrin to a high-affinity binding state (Wang et al. 2017). Experimentally, using single-molecule biomechanical approaches, Chen et al. (2024), compared force-modulated conformational changes in the ectodomain bending/unbending of integrins α5β1 and αVβ3. They observed distinctive mechanosensitivities in both integrins with various physiological implications. The α5β1 integrin rapidly switches between bent and extended conformations around the 7.4 pN threshold, allowing the cell to quickly detect extracellular stretch. This could enhance adhesion by recruiting and clustering more α5β1 integrins (Chen et al. 2024). On the other hand, the αVβ3 integrin exhibits bistability, with gradual conformational equilibrium shifts over a wide force range. This suggests that each αVβ3 molecule could act as a ‘biological dynamometer’, enabling the cell to ‘measure’ extracellular stretch force and local matrix stiffness (Chen et al. 2024).

For an αVβ3 integrin to form a strong, resilient mechanical bond with the RGD loop, it needs to acquire three divalent metal ions organized precisely (Craig et al. 2004; Paladino et al. 2017). The aspartate (Asp) in the RGD peptide makes direct contact with one of these metal ions about 4 Å away in a site called ‘metal-ion-dependent adhesion site’ (‘MIDAS’) (Craig et al. 2004). Additionally, this MIDAS ion is flanked by two nearby metal ions located in the ‘ligand-associated metal-binding site’ (‘LIMBS’) and the ‘adjacent to MIDAS’ (‘ADMIDAS’). This arrangement ensures that RGD binding to integrins does not involve forming a deep binding pocket. Instead, a shallow dynamic crevice forms at the interface between the two integrin subunits (Craig et al. 2004; Paladino et al. 2017).

In conclusion, the outside-in integrin activation mechanism is a highly regulated process involving conformational changes in these proteins, triggered by signals from the extracellular microenvironment. Detailed understanding of this mechanism could be crucial for comprehending processes like cell adhesion and migration.

##### Inside-out activation mechanism

Focal adhesions, multiprotein complexes formed at cell–ECM adhesion sites, act as mechanosensors, detecting and transmitting mechanical signals from the ECM to the cell interior (Shemesh et al. 2005). These structures serve as primary interfaces where extracellular mechanical signals intersect with intracellular biochemical signalling pathways and the cytoskeleton, being critical sites for mechanotransduction from both the outside-in and inside-out (Seong et al. 2013). Thus, extrinsic mechanical signals are detected through focal adhesions, influencing intracellular physiological functions, but intracellular mechanical signals can also be transferred to the extracellular microenvironment through focal adhesions to control the extracellular environment (Parsons et al. 2010). As such, bidirectional mechanotransduction can be transmitted through integrins in focal adhesions to regulate intracellular signalling or the ECM.

When forming focal adhesions, integrins connect the cell to the ECM through adaptor proteins such as α-actinin, vinculin, talin, paxillin and zixin (Geiger et al. 2009; Zaidel-Bar et al. 2007). In this way, they establish not only ligand-binding functions but also transmembrane connections to the cytoskeleton that activate intracellular signalling pathways (Kechagia et al. 2019; Kong et al. 2009). The described molecular couplings, although highly dynamic, exhibit sufficient stability to serve as a means of transmitting tensions from the ECM to the cell interior (Wang et al. 2009).

The general mechanism of mechanotransduction in focal adhesions translates mechanical force into biochemical information through conformational changes that expose crucial sites of target molecules necessary for downstream signalling pathways (Vogel 2006). Specifically, focal adhesion proteins may experience mechanical stretching due to traction force generated by actomyosin on integrins bound to the extracellular matrix (Seong et al. 2013).

One of the first proteins recruited during the biochemical activation of integrins is focal adhesion kinase (FAK) (Michael et al. 2009). FAK autophosphorylation promotes its activation, with this modification considered a trigger for intracellular mechanotransduction (Lachowski et al. 2018). Furthermore, the interaction between FAK and the contractile cytoskeletal network is regulated to maintain tension at specific sites and transfer force to the nucleus (Zhou et al. 2015).

Another key protein in the intracellular activation of integrins is talin, which binds to the cytoplasmic tail of the integrin β subunit. This interaction, in turn, causes the separation of the cytoplasmic domains of the α and β subunits, inducing their opening and triggering their bending and extension (Lu et al. 2022). Additionally, talin is notable for its nanomechanical properties, acting as a force buffer and defining the physiological force range that determines the mechanical stability of adhesions. This is due to its 13 C-terminal rod mechanosensitive subdomains, which exhibit gradual transmission kinetics (Yao et al. 2016). Thus, applying a stretch force of 2–12 pN unfolds talin, exposing vinculin binding sites (VBS) (del Rio et al. 2009). This exposure promotes the activation of a vinculin molecule. Similarly, all rod subdomains are mechanically vulnerable. This means that as the force applied to talin increases, more bundles unfold, exposing more VBS, and thus activating a greater number of vinculin molecules (Haining et al. 2016). This binding process is also crucial for promoting stable but flexible actin bundles and thus transferring the mechanical signal to the cell interior (Boujemaa-Paterski et al. 2020).

Functionally, short-term mechanotransduction events in focal adhesions can control cell expansion, morphology and migration. In the long term, these processes also regulate gene expression, cell differentiation and the development of conditions such as cancer (Nelson and Bissell 2006). Therefore, these mechanotransduction processes must be strictly regulated to maintain cell homeostasis (DuFort et al. 2011).

#### Cell–cell adhesion receptors: cadherins and icams

The primary architectural proteins in cell–cell junctions in all soft tissues are protein complexes associated with classical cadherins. Cadherin, a single-pass transmembrane protein, is widely recognized as a key molecule in cell–cell adhesion (Barry et al. 2014). These adhesions play a crucial role in resisting various forces within and around cells, including intracellular contractile tension, protrusive membrane forces and external pressures. Like integrins, cadherins are linked to actin through catenins, and the tension across adherens junctions is regulated by actomyosin-mediated contractility (Conway et al. 2013). Specifically, the cytodomain of cadherins forms a complex with β-catenin and α-catenin. The latter is in an autoinhibited state under basal conditions (Yonemura et al. 2010), but an increase in tension of about 5 pN relieves this state, inducing a conformational change that exposes VBS. This exposure leads to the recruitment of vinculin, marking the initiation of mechanotransduction (Yao et al. 2014).

The bonds formed by cadherins inherently transmit mechanical signals to cells by resisting forces generated internally or externally, such as fluid shear stress, tissue rigidity, compression or tension. The response of cadherin complexes to internal and external tensions indicates their role as mechanosensors (Muhamed et al. 2017). While cadherin ligation alone can trigger biochemical signalling, the connection of cadherin complexes to adjacent cellular cytoskeletons forms a mechanical chain that experiences fluctuations in traction forces due to dynamic cytoskeletal changes. Additionally, different types of cadherin, such as C-cadherin, N-cadherin, E-cadherin and VE-cadherin, exhibit local junctional remodelling dependent on actin and increased stiffness following disruptions in cadherin receptor interactions (Barry et al. 2014; Tabdili et al. 2012). These deformations in cytoskeletal components are vital in processes such as embryonic tissue development (morphogenesis), tissue repair and diseases like cancer (Leckband and de Rooij 2014).

On the other hand, ICAMs are membrane proteins that also play an important role in cell–cell adhesion and communication. These proteins are ligands for immunologically significant integrins present on inflamed endothelium and antigen-presenting cells (Takada et al. 2007), with specific mechanisms involved in mechanotransduction still under active investigation. Among the most studied is ICAM-1. In inflamed endothelial tissue, neutrophils displaced through selectins induce the release of calcium from the endoplasmic reticulum reservoir. This acts synergistically with various chemokines, promoting the formation of high-affinity bonds between LFA-1 and ICAM-1, necessary to anchor cells against the force of blood flow (Morikis et al. 2020). Additionally, the role of ICAM-1 as a biosensor should be highlighted, as it integrates signals from the exterior and transmits them to the cell interior through the association of its cytoplasmic domain with the actin cytoskeleton (Bui et al. 2020; Mahato et al. 2020; Uday Pratap Azad 2023). Indeed, it is this association that strengthens adhesion, allowing cells to exert higher traction forces for faster migration. This is because shear forces cause rearrangements in actin filaments and promote the nano-clustering of ICAM-1 molecules, ultimately increasing the number of ICAM-1/LFA-1 bonds (Piechocka et al. 2021).

Another important adhesion molecule is platelet/endothelial cell adhesion molecule-1 (PECAM-1). This transmembrane adhesion receptor converts shear forces derived from blood flow into endothelial cell alignment in the direction of flow (Tzima et al. 2005). On the other hand, it has been shown that disturbed shear stress activates nuclear factor-kappa B (NF-κB) and inflammatory responses through PECAM-1, initiating atherogenesis and vascular remodelling (Chen and Tzima 2008). Additionally, in regions of disturbed shear stress, a stiffer cellular phenotype develops. In this context, PECAM-1 may be involved as force applied to this molecule activates PI3K, which promotes global integrin activation and subsequent activation of the GTPase RhoA through LARG. This induces cytoskeletal changes, leading to adaptive stiffening and increased focal adhesions (Collins et al. 2012).

In summary, cadherins and ICAMs are essential for cell–cell adhesion and communication in soft tissues. Cadherins, linked to actin through catenins, resist various forces and act as mechanical sensors, crucial for processes such as tissue development, healing and cancer. ICAMs, especially ICAM-1 and PECAM-1, play important roles in immune responses and cell alignment in blood flow, contributing to vascular remodelling and cytoskeletal stiffening.

### Distal mechanotransduction

Traditionally, the cell nucleus has been regarded as the passive epicentre of genetic information. Currently, a paradigm shift is emerging that positions this organelle as a central effector in mechanosensory processes, where its tensile state acts as a dynamic sensor of cellular compression and stretching. These processes involve the participation of essential proteins of the nuclear envelope (NE), such as lamin A/C and emerin, and multiprotein assemblies like the LINC complex, which regulate the structure and function of the NE. The transduction of mechanical signals through these proteins triggers biochemical and molecular responses that significantly impact cellular physiology.

#### LINC complex

**Fig. 4:**
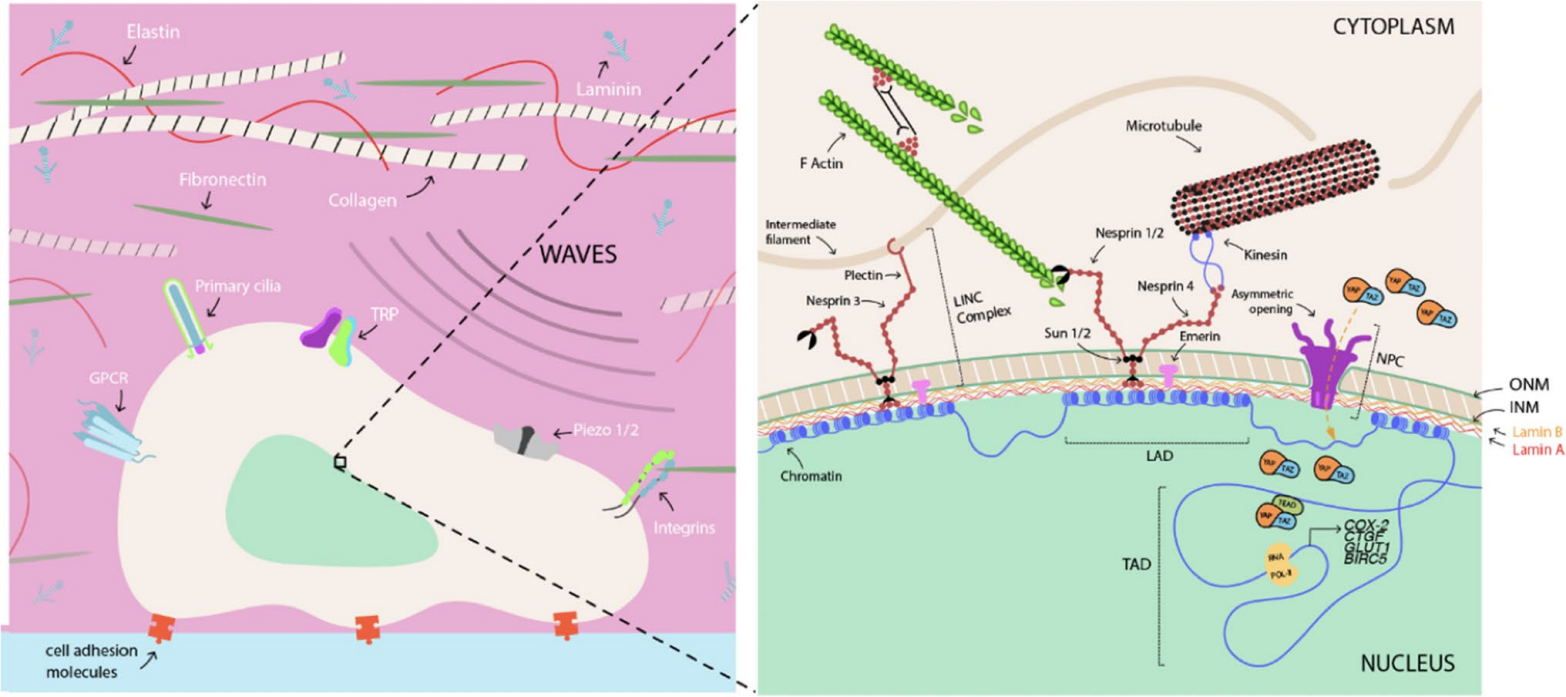
Nuclear envelope composition and nucleoskeleton interactions. LINC complexes promote physical coupling between the cytoskeleton and the interior of the nucleus, partially balancing the cytoskeletal traction force exerted on the outer nuclear membrane (ONM). Likewise, the tension produced on the nuclear membrane induces the opening of the nuclear pore complex (NPC) in an asymmetric manner, facilitating the entry of the yes-associated protein/transcriptional co-activator with PDZ-binding motif (YAP/TAZ) co-activator into the nucleus, where it performs its function. Furthermore, chromatin undergoes changes due to its interaction with the nuclear lamina through the lamina-associated domains (LADs), promoting the expression of specific genetic programs that help the cell maintain its homeostasis and mechanoprotection of the genome. *TAD* topologically associated domains, *INM* inner nuclear membrane

Once inside the cell, nuclear envelope proteins are required for biophysical signals to reach the nucleus. One of the most studied systems is the linker of nucleoskeleton and cytoskeleton (LINC) complex (Lele et al. 2018; Uhler & Shivashankar 2017). LINC complexes consist of nesprins, SUN proteins and lamins (Fig. 4) (Crisp et al. 2006; Zhang et al. 2007). In detail, proteins of the KASH domain located in the outer nuclear membrane extend both towards the cytoplasm, interacting with elements of the cytoskeleton (actin, microtubules and intermediate filaments) and towards the perinuclear space, where they bind to SUN domain proteins (Spindler et al. 2019). Simultaneously, SUN domain proteins traverse the perinuclear space (approximately 50 nm thick) and cross the inner nuclear membrane to reach the nucleus (Crisp et al. 2006). These proteins can interact not only with the nuclear lamina (via SUN1 and SUN2) but also with chromatin (Gundersen and Worman 2013). On the outer nuclear membrane, nesprin 1 and nesprin 2 connect SUN1 (also UNC84A) via actin microfilaments; SUN2 (also UNC84B) connects to the inner nuclear membrane; SUN1 binds to lamin A in the nuclear structure (Chang et al. 2015; Lee and Burke 2018).

The nuclear lamina is a network composed of integral membrane proteins (emerin, lamin-associated polypeptide 2 and MAN1) and type V nuclear intermediate filaments (lamins A, B and C), which provide mechanical support to the inner nuclear membrane (Barton et al. 2015; de Leeuw et al. 2018). These lamins can directly connect with the genetic machinery and DNA or through binding to other nucleoproteins (emerin or lamin B receptor), influencing relevant aspects such as epigenetics, chromatin organization, DNA replication, transcription and DNA repair (Bickmore and Van Steensel 2013; Lele et al. 2018). Additionally, emerin is part of the LINC complex, connecting the nuclear lamina to the actin cytoskeleton and participating in chromatin modification, transcriptional regulation and mRNA processing (Holaska and Wilson 2007; Salpingidou et al. 2007).

The LINC complex has various functions, including nuclear migration, maintenance of nuclear morphology and positioning, centrosome–nucleus interaction, DNA repair, cell migration, and intranuclear chromosomal movement during meiosis (Bone and Starr 2016; Chancellor et al. 2010). Additionally, signal transmission by the LINC complex occurs bidirectionally, i.e. from the cell surface to the nucleus as well as from the nucleus to the cytoplasm (Chambliss et al. 2013; Schwartz et al. 2017).

##### Signalling from outside in through the LINC complex

Numerous examples demonstrate the existence of mechanotransduction pathways to the cell nucleus through the LINC complex. In terms of cell proliferation, cyclic equibiaxial stretching inhibits differentiation and induces proliferation of C2C12 cell line. However, overexpression of SUN1 protein constructs or dominant-negative nesprin-2 negates stretch-induced proliferation (Brosig et al. 2010). The absence of KASH or depletion of nesprin-2G or nesprin-3 proteins from the LINC complex has been observed to inhibit perinuclear actin cap assembly (Chambliss et al. 2013). Furthermore, focal adhesions associated with this perinuclear actin cap (ACAFA) are different in shape, size and spatial distribution compared to conventional focal adhesions (Chambliss et al. 2013). Another study demonstrated that using magnetic tweezers that force application on nesprin-1 triggers local nuclear stiffening that does not involve chromatin or nuclear actin but requires intact nuclear lamina and emerin. Because of nesprin-1 traction, emerin undergoes Src tyrosine kinase–dependent phosphorylation, reinforcing the interaction between lamin A/C and the LINC complex, which is relevant for the expression of mechanosensitive genes (Guilluy et al. 2014). These findings indicate an interconnected physical pathway involving the cytoskeleton–LINC complex, allowing transmission of mechanical signals from the extracellular environment to the nucleus.

##### Signalling from inside out through the LINC complex

There are multiple mechanisms through which the LINC complex sends signals from the nuclear envelope to critical regulators of the actin cytoskeleton. LINC complexes containing the SUN2 protein promote focal adhesion (FA) assembly by activating the small GTPase RhoA through a transcription-independent mechanism. Conversely, the SUN1 protein has an antagonistic function, inhibiting RhoA activation and FA assembly (Thakar et al. 2017). Myoblasts with mutations Nespr-1^ΔKASH^ and LMNA^ΔK32^ have been observed to impair adaptation to their mechanical environment within a range of stiffness encompassing muscle tissue (Schwartz et al. 2017). On soft substrates, these mutant precursor cells exhibited accumulation of contractile actin stress fibres, increased traction force and thinner nuclei compared to controls. Inhibition of Rho-associated kinase (ROCK) or inactivation of a ROCK-dependent actin remodelling regulator, formin FHOD1, largely restored morphology in mutant myoblasts. These results underscore the need for functional integrity of lamin and nesprin-1 to modulate FHOD1 activity and inside-out mechanical coupling that adjusts the cell’s internal stiffness to match that of its soft and physiological environment (Schwartz et al. 2017). Finally, Infante et al. (2018) demonstrated that an intact connection between the nucleus and the centrosome through nesprin-2 and the dynein adapter Lis1 is required to regulate MT1-MMP trafficking. This metalloproteinase, involved in pericellular collagenolysis, migrates from intracellular storage compartments in front of the nucleus to the cell surface, enabling confined migration in tumour cells (Infante et al. 2018). These findings indicate diverse pathways through which the LINC complex sends signals to the cytoplasm, promoting a multitude of adaptation and survival strategies.

In summary, LINC complexes can function as bidirectional integrative centres in mechanotransduction, as the physical connection between the cytoskeleton and the LINC complex provides mechanical coupling, making the cell, cytoskeleton and nucleus function as a whole and enabling the propagation of mechanical energy (Song et al. 2020; Wang et al. 2009). Additionally, LINC complexes possess a multimodular structural capacity. Considering only the two most widespread SUN proteins in mammals (SUN1 and SUN2) and the six KASH proteins, hundreds of possible LINC complexes are obtained, allowing multiple levels of regulation (McGillivary et al. 2023); enabling the involvement of biosignals in critical functions related to nuclear structure, shape and position (Alam et al. 2015; Luxton et al. 2010); and affecting gene expression and transcription (Tajik et al. 2016; Wang, Ning et al. 2009).

#### Yes-associated protein/transcriptional co-activator with PDZ-binding motif

Beyond the LINC complex, the yes-associated protein/transcriptional co-activator with PDZ-binding motif (YAP/TAZ) proteins act like a nuclear switch, toggling between on and off states (Dupont et al. 2011; Martino et al. 2018). These nuclear effectors of the Hippo pathway (Pan 2010) also participate in cellular mechanics and cytoskeletal dynamics, and contribute to stabilizing extracellular matrix structure (Fu et al. 2010; Wang et al. 2009). Additionally, YAP/TAZ controls the expression of focal adhesion proteins and actin regulators (Morikawa et al. 2015; Nardone et al. 2017). Moreover, they have been shown to contribute to storing ‘memory’ of past cell–ECM interactions (Yang et al. 2014).

Regarding cell survival, YAP/TAZ plays an ambivalent role in apoptosis. Their binding to transcriptional enhanced associate domain (TEAD) transcription factors promotes the expression of anti-apoptotic genes such as *COX-2*, *BIRC5* and *Glut1* (Fig. 4) (Zhang et al. 2018). However, under certain circumstances, when cells undergo DNA damage stress, the tyrosine kinase c-Abl enters the nucleus and phosphorylates YAP at a tyrosine residue, Y357. This increases its affinity with p73, allowing interaction and inducing the transcription of various pro-apoptotic genes such as *p53AIP1*, *Bax*, *DR5* and *PUMA* (Cheng et al. 2022; Zhang et al. 2018). Another mechanism in which YAP/TAZ is involved is iron-dependent regulated cell death or ferroptosis, through the positive regulation of various ferroptosis modulators such as *ACSL4* and *TFRC* (Wu et al. 2019).

This multiplicity of functions is regulated by a complex network of molecular interactions whose upstream factors can be classified into five major groups: soluble factors, cell density, stress signals, cell polarity and mechanical signals (Fu et al. 2022).

##### Regulation of YAP/TAZ dependent on the Hippo pathway

The Hippo pathway is an evolutionarily conserved on/off signalling route involved in numerous biological processes such as organogenesis and tissue homeostasis (Chang et al. 2020). At the molecular level, the core of the pathway is a kinase cascade, where MST1/2 (serine/threonine kinase 4), SAV1 (Salvador family WW domain containing protein 1), LATS1/2 (large tumour suppressor kinase 1/2), YAP and TAZ are considered key components (Fu et al. 2022). Typically, the striatin-interacting phosphatase and kinase complex (STRIPAK) functions upstream of MAP4K (mitogen-activated protein kinase kinase kinase kinase) and MST1/2 and inhibits the Hippo pathway. However, upon pathway activation, MAP4K, MST1/2 and its scaffold protein SAV1 phosphorylate LATS1/2 and its scaffold MOB1A/B (MOB kinase activator 1A/B). Subsequently, YAP and TAZ are phosphorylated and inhibited by LATS1/2, retaining them in the cytoplasm, preventing their nuclear translocation and interaction with TEAD family members (Chang et al. 2020; Fu et al. 2022; Ma et al. 2019; Misra and Irvine 2018).

Through the Hippo pathway, cells can detect a multitude of mechanical signals such as cell–ECM and cell–cell contacts, ECM stiffness, fluid shear, cell geometry and actomyosin internal tension (Dasgupta and McCollum 2019). For example, with low ECM stiffness, the GTPase RAP2 is activated and stimulates various kinases such as MAP4K4 and MAP4K6, leading to the activation of LATS1 and LATS2 and inhibition of YAP and TAZ (Meng et al. 2018). Additionally, RAP2 has been linked to cytoskeletal dynamics, positioning it as a central integrator of cytoskeletal signals for Hippo pathway signalling (Wu et al. 2023). On the other hand, cytoskeletal tension and cell–cell contacts regulate the Hippo pathway through various proteins such as LIMD1 (LIM domain–containing protein 1) and Rho. LIMD1 is found in adherens junctions under conditions of cytoskeletal tension, low cell density and Rho activation dependence. In this context, LIMD1 recruits LATS kinases and inhibits their function, silencing the Hippo pathway (Ibar et al. 2018). Furthermore, there are other proteins that connect the cytoskeleton and the Hippo pathway, such as angiomotin-130 (AMOT130). This protein contains a conserved binding domain that can interact with both F-actin and YAP. AMOT130 undergoes LATS-dependent phosphorylation, preventing it from binding to F-actin. However, this modification increases the affinity for YAP, resulting in an AMOT130–YAP interaction complex, retaining YAP in the cytoplasm (Mana-Capelli et al. 2014).

##### Non-canonical mechanoregulation of YAP/TAZ

Although there are many upstream signals regulating the function of YAP/TAZ through their phosphorylation via the Hippo pathway, there are multiple mechanisms that regulate the stability, activity and subcellular localization of these co-activators independent of the canonical pathway (Cho and Jiang 2021). For example, the loss of the plasma membrane protein caveolin-1 (CAV-1) is associated with an increase in phosphorylated YAP, possibly through a mechanism protecting against dephosphorylation via interaction with the protein 14–3-3YWHAH. This facilitates the cytoplasmic sequestration of YAP in an F-actin-dependent manner and independent of hypokinase. Therefore, CAV-1 emerges as a positive regulator upstream of YAP, affecting the adaptive response to changes in ECM stiffness by YAP (Moreno-Vicente et al. 2018).

Also, the nucleocytoplasmic transport of YAP is not only stimulated by biochemical signals such as post-translational modifications. An increase in cellular tension is associated with mechanosensitive nuclear import of YAP. Under basal conditions, the internal light of nuclear pores comprises a flexible disorganized network of proteins containing phenylalanine–glycine-rich repeats, impairing the free diffusion of molecules and exerting mechanical resistance. However, during mechanotransmission, due to both vertical and lateral internal compression forces, nuclear deformation occurs (Elosegui-Artola et al. 2017). This distortion increases the permeability of nuclear pore complexes (NPCs), as their diameter asymmetrically enlarges. This reduces mechanical restriction to passage and increases nuclear membrane curvature, favouring YAP translocation into the nucleus independent of the Hippo pathway. Additionally, the low mechanical stability of YAP due to its structurally disordered regions would facilitate the process (Elosegui-Artola et al. 2017). Once inside the nucleus, protein–protein interactions play a fundamental role in the mechanoregulation of YAP. In situations of low mechanical signalling, the ARID1A–SWI/SNF complex interacts with YAP/TAZ, inhibiting the possibility of interaction between YAP/TAZ and TEAD 1–4. Conversely, under high mechanical stress, nuclear F-actin binds to ARID1A–SWI/SNF, preventing the formation of the ARID1A–SWI/SNF–YAP/TAZ complex in favour of the interaction between YAP/TAZ and TEAD 1–4 (Chang et al. 2018). Based on these considerations, various cytoskeletal components can regulate YAP/TAZ at multiple levels, modulating both their intracellular compartmentalization and functionality.

Actomyosin contractility facilitates the nuclear translocation of YAP/TAZ in various cell types. However, evidence suggests that there is a higher level of difficulty in mechanoregulating the subcellular localization of YAP/TAZ based on actomyosin tension. While cells in a low-confluence state present large stress fibres connecting to focal adhesions, highly confluent epithelial cells mainly generate actomyosin filaments associated with adherens junctions (AJs). Actomyosin-based traction force at AJs induces the translocation of Merlin from AJs to the nucleus, which interacts with YAP. Once the Merlin–YAP complex is formed, it is exported to the cytoplasm, mediated by Merlin’s nuclear export signals (NES) (Furukawa et al. 2017). Thus, it is suggested that actomyosin activity has opposing effects on the subcellular distribution of YAP depending on the cellular context.

In summary, the mechanosensitive co-activator YAP/TAZ emerges as a key regulator in cells’ ability to integrate different mechanical signals from the cellular microenvironment and translate them into specific adaptive biological responses. Their complex regulation involves the participation of signalling pathways such as the Hippo pathway and other non-canonical pathways, such as cytoskeletal tension, affecting different aspects such as the stability, activity and subcellular localization of YAP/TAZ depending on the cellular context.

#### Chromatin

The interior of the cell nucleus, primarily composed of chromatin (euchromatin or transcriptionally active chromatin and heterochromatin or transcriptionally inactive chromatin) and nuclear bodies such as nucleoli and Cajal bodies, exhibits fundamental physical and organizational properties for the integrity and response of the nucleus to mechanical forces. In this context, physical connections between chromatin and the nuclear envelope contribute to nuclear stiffness and stability, as well as the regulation of gene expression (Kind and van Steensel 2010; Peric-Hupkes and van Steensel 2010).

##### Gene mechanoregulation

A crucial factor defining the mechanical properties of the nucleus and crucial for regulating gene expression is the degree of chromatin compaction (Schreiner et al. 2015). In eukaryotic cells, the total length of DNA is approximately 2 m, which is wrapped around histones with the assistance of non-histone proteins (Gilbert et al. 2005). Considering that the typical size of a eukaryotic nucleus falls within the range of 5–20 µm (Lammerding 2011), it is expected that chromatin exerts entropic pressure outward on the nuclear envelope, influencing nuclear stiffening. It follows that changes in chromatin compaction directly influence nuclear rigidity (Furusawa et al. 2015). Chromatin decompaction decreases anisotropic nuclear deformations caused by constant force application on fibroblast nuclei (Haase et al. 2016). Therefore, increased euchromatin formation leads to less rigid nuclei (Furusawa et al. 2015). Moreover, the control of nuclear stiffness exerted by the euchromatin/heterochromatin ratio appears to depend on kinetic–spatial parameters, specifically less than 3 µm stretching at a stretching rate of 50 nm/s in isolated HeLa cell nuclei (Stephens et al. 2017).

The mechanism by which the nucleus regulates chromatin dynamics has commonly been interpreted under the conceptual framework of chromatin interaction with nuclear envelope proteins (Kind and van Steensel 2010; Reddy et al. 2008). However, the influence of this binding on the overall three-dimensional organization of chromatin remains poorly understood. Several studies have shown that nuclear lamina anchors lamina-associated domains (LADs) at the nuclear periphery (Fig. 4) (Chang et al. 2022; Zheng et al. 2018). Furthermore, they are responsible for maintaining proper interactions between topologically associated domains (TADs), as well as transcriptional states, through the maintenance of active and inactive chromatins (Zheng et al. 2018). In the case of negative regulation of lamin A/C, such as in fibroblasts coated with isotropic shapes, reduced connection between chromosomes and the inner nuclear membrane causes differential chromosomal repositioning, resulting in the formation of new chromosomal environments and neighbourhoods (Wang et al. 2017). Additionally, cardiomyocyte differentiation is associated with the reorganization of peripheral heterochromatin, with the histone deacetylase protein Hdac3 necessary for binding peripheral heterochromatin to the nuclear lamina. Hdac3 removal in cardiac progenitor cells releases genomic regions from nuclear periphery, leading to early expression of cardiac genes and their differentiation into cardiomyocytes (Poleshko et al. 2017). Furthermore, among structural chromatin proteins, CTCF, associated with genome architecture regulation and maintenance of genome accessibility regions, plays a crucial role. Suppression of this protein, enriched in LAD and TAD boundaries, significantly decreases long-range interactions of cardiac gene enhancers, ultimately leading to cardiomyopathy development (Rosa-Garrido et al. 2017). Consequently, mechanical forces causing conformational changes in the nuclear lamina may have a significant impact on how chromatin is organized, thereby affecting gene mechanoregulation in different cell types.

Another crucial aspect is the mechanoregulation of transcription factors. For example, culturing fibroblasts on a polarized surface induces the expression of genes related to the cytoskeleton and ECM, due to the action of myocardin-related transcription factor (MRTF). MRTF is a transcriptional co-factor found bound to G-actin. With increased stiffness, the formation of F-actin stress fibres is promoted, causing G-actin to dissociate from MRTF, allowing the latter to be imported into the nucleus. In contrast, when fibroblasts are on isotropic surfaces, expression of cell cycle–related genes increases via the NF-κB pathway, mediated by p65 (Jain et al. 2013). Furthermore, both pathways seem to exhibit crosstalk orchestrated by alterations in actomyosin contractility, as decreased actin polymerization results in p65 translocation into the nucleus, while MRTF is exported to the cytoplasm (Tang et al. 2008). This highlights the existence of mechanical control mechanisms of gene expression through differential nuclear localization of transcription factors.

##### Mechanoprotection of chromatin

Nuclear deformation without nuclear envelope rupture can cause DNA damage, primarily during active replication. This damage is related to replication stress, possibly due to torsional stress on DNA generated in this situation (Shah et al. 2021). Besides, subjecting epidermal progenitor cells (EPCs)/stem cells to cyclic uniaxial stretching, they attempt to counteract the effect of nuclear deformation by calcium-dependent nuclear softening mediated by Piezo1 release from the endoplasmic reticulum. This results in the loss of heterochromatin marked with H3K9me3 associated with the lamina (Nava et al. 2020). Furthermore, although chromatin exhibits solid-like properties at the mesoscale, in the presence of high concentrations of cations, it can undergo a liquid–liquid phase separation process and locally behave like a condensate of separate phases (Ng et al. 2022). This fluidification of chromatin provides an efficient means to dissipate mechanical energy (Bonakdar et al. 2016), thereby reducing direct force propagation to DNA, avoiding possible torsions and/or breaks. Therefore, changes in chromatin rheology and architecture can also serve as mechanoadaptive mechanisms to prevent potential DNA damage under high tension (Nava et al. 2020).

Broadly, the mechanoregulation of gene expression emerges as a multidimensional phenomenon, where different mechanical signals reaching the nucleus induce changes in chromatin organization, as well as activation of various metabolic pathways, converging in the creation of specific gene expression programs. Additionally, cells possess various mechanoprotective mechanisms that attempt to counteract potential DNA damage induced by mechanical stress, ensuring their survival.

## Challenges and perspectives in sonobiology and biomedicine

Mechanotransduction and sonobiology have emerged as key areas within biomedicine, opening new possibilities for both the diagnosis and treatment of various pathologies. However, their implementation faces several significant challenges. One of the main issues lies in the lack of methodological standardization, which compromises the reproducibility of results and creates a gap between findings in basic research and their clinical application. Therefore, the standardization of protocols and methods becomes a priority to facilitate progress in this field.

Moreover, mechanotransduction involves complex cellular responses due to the interconnection of multiple pathways and mechanisms. Fully characterizing these phenomena and translating them into clinical practice, for both diagnosis and therapy, poses a considerable challenge from a scientific and technical perspective. Added to this is the heterogeneity of tissue microenvironments. As highlighted in the research, the extracellular context is crucial to mechanosensitive processes. However, tissues vary in terms of stiffness, elasticity and structural organization, meaning that acoustic waves and other mechanical forces may be perceived differently across various tissue types. This complicates the development of uniform approaches applicable to complex tissue systems, such as tumours.

On the other hand, sonobiology, particularly in its application to regenerative therapies and cancer treatments, must meet strict regulatory requirements. Ensuring long-term safety, minimizing adverse effects and demonstrating efficacy across diverse patient populations are aspects that demand innovative solutions for the clinical implementation of these technologies.

In this context, despite the wide variety of biological mechanisms that have been investigated in the mechanotransduction process, the specific effects of AAWs and LVS on mammalian cells remain largely unexplored. Understanding the mechanisms by which AAWs and LVS influence cellular behaviour, tissue responses and physiological processes could promote more effective therapeutic interventions, more accurate diagnostics and more advanced medical technologies. Regarding biomedical applications, AAWs and LVS stimulation have potential applications in numerous fields ranging from dentistry and wound care to bone health, tissue engineering and cancer research (Kennedy et al. 2021; Mohammed et al. 2016). For example, in the field of dental research, sound waves could promote the proliferation of gingival fibroblasts (Jones et al. 2000). Likewise, the implications of AAW-induced neural differentiation for tissue engineering are promising, paving the way for the development of new strategies for tissue regeneration and repair (Choi et al. 2016). In the context of cancer research, AAW stimulation could optimize the biofabrication of extracellular EVs from cancer cells, representing a novel path with implications for cancer diagnosis and therapy (Lei et al. 2023).

Looking ahead, the convergence of these disciplines offers exciting prospects for interdisciplinary collaboration and translational applications. It is imperative to further explore the multidimensional applications of acoustic stimulation across the audible spectrum and to foster synergies between fields to unlock the full potential of AAW approaches for clinical and therapeutic innovation. Possible future research could explore the use of AAW stimulation in conjunction with other differentiation inducers or scaffolds to enhance functional tissue maturation. At the same time, further studies are warranted to investigate the potential use of AAWs to generate EVs from various cell lines, including stem cells. This research could shed light on its importance in the fields of regenerative medicine and tissue engineering, and explore AAW-enhanced clinical implications in cancer detection, prognosis and diagnosis.

## Conclusions

When an AAW hits a culture plate, part of the energy induces structural vibrations leading to the generation of LVS, and part of the energy is transferred to the interior of the plate to interact with the biological material. The biophysical study of wave–cell and wave–structure interactions is facilitated by mechanotransduction mechanisms in the field of sonobiology. Taken together, the current research demonstrates the potential of LVS and AAW on mammalian cell behaviour and emphasizes the determining role of specific physical parameters during stimulation, such as frequency, sound pressure level/amplitude and exposure time. Furthermore, the analysed results highlight the impact of vibroacoustic stimulation on key biological processes such as cell proliferation, differentiation, protein production, gene expression and cytoskeletal dynamics. After a thorough analysis of the biological pathways and agents involved in mechanotransduction within the field of sonobiology, the following conclusions can be drawn:

(i) Stress-activated membrane ion channels play vital roles in cell survival and development. Recent studies suggest that integrins may utilize these channels for physiological control. Additionally, mechanosensitive ion channels, like TRPV4, regulate cellular behaviours such as focal adhesion assembly and directional migration in response to mechanical strain. Moreover, the activation of other channels such as Piezo1 leads to the influx of Ca^2+^ cations, triggering biochemical signalling pathways such as PI3K/Akt and MT1-MMP, involved in processes such as vascular remodelling and angiogenesis.

(ii) Key ECM molecules like fibronectin and integrins, the primary cell–matrix adhesion proteins, facilitate the ECM–membrane interface by forming focal adhesions. These adhesions, linked to the cytoskeleton, serve as mechanosensors, translating extracellular mechanical cues into intracellular biochemical signals. Bidirectional mechanotransduction occurs through integrins at focal adhesions, influencing cellular functions and gene expression, with tight regulation essential for cellular homeostasis.

(iii) Cadherins, crucial architectural proteins at cell–cell junctions, mediate adhesion and resist internal and external forces through interactions with the cytoskeleton. Linked to actin via catenins, cadherins transmit mechanical signals, acting as mechanosensors responsive to various stresses like shear stress and tissue stiffness. Their role extends to processes such as embryonic morphogenesis, tissue healing and disease progression, highlighting their significance in cellular physiology and pathology. Similarly, we find intercellular adhesion molecules, which are directly related to the immune response, promoting vascular remodelling and cytoskeletal stiffening.

(iv) LINC complexes operate as bidirectional integrative centres for mechanical signals at multiple levels. Firstly, the physical coupling provided by the LINC complex between the cytoskeleton and the nucleus allows the propagation of mechanical energy into the nucleus. Secondly, the structural versatility of LINC complexes enables the generation of specific cellular responses based on integrated biomechanical signals. As a result, this structure is involved in multiple vital cellular functions, such as cell migration, DNA repair and intranuclear chromosomal movement during the process of meiosis, among other processes.

(v) The mechanosensitive co-activator YAP/TAZ acts as a central regulator in the mechanotransduction process, granting the cell the ability to integrate upward mechanical signals from the cellular microenvironment and translate them into adaptive biological responses. This is achieved through their complex mechanical and biochemical regulation, which affects their chemical characteristics and subcellular localization. Thanks to this, YAP/TAZ has a broad spectrum of functions within the context of cell survival, highlighting an ambivalent role in the processes of apoptosis and cell proliferation.

(vi) Mechanical biosignals reaching the nucleus can induce changes in chromatin dynamics, which, along with the mechanoregulation of various transcription factors, trigger specific gene expression programs. These changes in chromatin architecture offer the possibility of a cellular response but can also serve as a mechanoprotective mechanism by preventing potential DNA damage in response to increased intracellular tension. Additionally, this adaptive capacity should be considered as a direct consequence of its physicochemical properties, which favour the dissipation of mechanical energy under high tension.

Ultimately, sonobiology emerges as an approach with great potential to address global challenges in the healthcare sector, opening new possibilities in areas such as diagnosis, the development of new medical technologies and therapy. However, to ensure consistent development of this emerging field, it is vital to implement standardized measurement techniques and controlled, biocompatible experimental set-ups. All this will guarantee experimental reproducibility, facilitate the transfer of knowledge and promote the joint action of different fields of knowledge, such as physics, biology and engineering, in order to obtain an integral vision of how the human organism and the universe work.

## Data Availability

No datasets were generated or analysed during the current study.
